# Photoplethysmography temporal marker-based machine learning classifier for anesthesia drug detection

**DOI:** 10.1007/s11517-022-02658-1

**Published:** 2022-09-05

**Authors:** Syed Ghufran Khalid, Syed Mehmood Ali, Haipeng Liu, Aisha Ghazal Qurashi, Uzma Ali

**Affiliations:** 1 Faculty of Health, Education and Life Sciences, Birmingham City University, Birmingham, B15 3TN UK; 2 Biomedical Engineering Department, College of Engineering, Imam Abdulrahman Bin Faisal University, King Faisal Rd, Dammam, 34212 Saudi Arabia; 3 Research Centre for Intelligent Healthcare, Coventry University, Coventry, CV1 5FB UK; 4 General Medicine, Jubilee Health Care, Westminster Rd, Coventry, CV1 3GB UK; 5 Department of Public Health, College of Public Health, Imam Abdulrahman Bin Faisal University, King Faisal Rd, Dammam, 34212 Saudi Arabia

**Keywords:** Photoplethysmography, Anesthesia depth, K-nearest neighbor, Queensland database, MIMIC II database

## Abstract

Anesthesia drug overdose hazards and lack of gold standards in anesthesia monitoring lead to an urgent need for accurate anesthesia drug detection. To investigate the PPG waveform features affected by anesthesia drugs and develop a machine-learning classifier with high anesthesia drug sensitivity. This study used 64 anesthesia and non-anesthesia patient data (32 cases each), extracted from Queensland and MIMIC-II databases, respectively. The key waveform features (total area, rising time, width 75%, 50%, and 25%) were extracted from 16,310 signal recordings (5-s duration). Discriminant analysis, support vector machine (SVM), and K-nearest neighbor (KNN) were evaluated by splitting the dataset into halve training (11 patients, 8570 segments) and halve testing dataset (11 patients, 7740 segments). Significant differences exist between PPG waveform features of anesthesia and non-anesthesia groups (*p* < 0.05) except total area feature (*p* > 0.05). The KNN classifier achieved 91.7% (AUC = 0.95) anesthesia detection accuracy with the highest sensitivity (0.88) and specificity (0.90) as compared to other classifiers. Kohen’s kappa also shows almost perfect agreement (0.79) with the KNN classifier. The KNN classifier trained with significant PPG features has the potential to be used as a reliable, non-invasive, and low-cost method for the detection of anesthesia drugs for depth analysis during surgical operations and postoperative monitoring.

**Graphical abstract:**

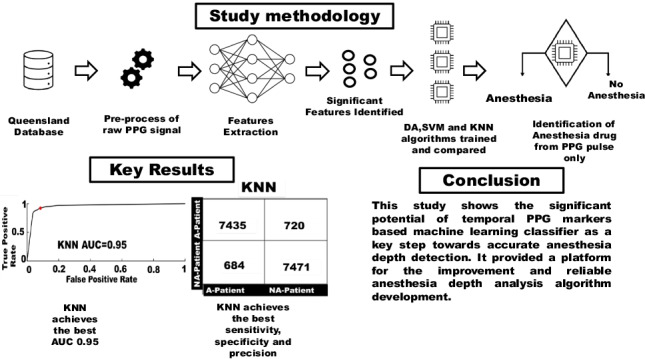

## Introduction

General anesthesia causes a total or partial loss of sensation and anesthesia drugs affect pulmonary and cardiovascular systems [1, 2]. The autonomic nervous system (ANS) controls the autonomous activity during the unconsciousness of the patient. Stimulation during surgical procedures causes changes in cardiac and pulmonary systems by ANS [3]. At least more than one type of anesthesia drug is used for the patients. Patients with cardiovascular diseases (i.e., myocardial infarction, hypertension) are more susceptible to anesthesia complications. Overdosing of anesthesia drugs can cause severe complications that could be life-threatening. Related vital signs (e.g., heart rate, respiratory rate) are often monitored during anesthesia. Anesthesia depth has been detected by the electroencephalographic modalities that include the bispectral index and spectral entropy but these modalities are expensive and insensitive to different anesthetic drugs [4]. Due to its clinical importance, recently, some novel technologies based on electroencephalography have been proposed for detecting anesthesia depth [5]. A deep learning-based algorithm using electrocardiography (ECG) and photoplethysmography (PPG) was proposed but two-channel analysis has the potential to be complicated during implementation [6]. Therefore, there is an urgent clinical need for a simple, non-invasive, low-cost, and reliable method to detect anesthesia depth.

PPG is a low-cost, non-invasive technology widely used for blood saturation and heart rate measurement [7]. PPG waveform reflects the volumetric changes in the distal circulation and is influenced by the respiratory drive and other patient-specific physiological conditions. Earlier research revealed that the time-based PPG waveform features are associated with cardiovascular changes in the human body [8]. Recently, PPG has been widely used in extracting cardiovascular and respiratory parameters [8–10]. PPG can reflect the vital signs related to the anesthesia depth [8–10]. Therefore, recent years have witnessed increasing works on PPG-based anesthesia depth detection [12–14].

PPG waveforms have been used to investigate the depth of anesthesia or to validate other technologies during surgical operations or post-surgical procedures [10–14]. Coutrot et al. examined the detection of intraoperative hypotension by the PPG waveform features (dicrotic notch height (DIC) and perfusion index (PI)) during vasopressor boluses [11]. Both parameters separately achieved a good area under the curve (AUC) of 0.86 and 0.83 and the combination of both parameters improved prediction better with an AUC of 0.91 [12]. Ezri et al. and Chen et al. both used digital and analogue finger PPG waveforms to detect and classify anesthesia depth whilst different parameters were used (PPG amplitude in Ezri et al.’s study, approximate and sample entropies in Chen et al.’s study with AUC of 0.87) [12, 13]. Bao used various PPG waveform features (amplitude, notch, baseline amplitude, and area) to achieve a balanced anesthesia detection. The PPG waveform features and cerebral state index were used to compare the detection of balanced anesthesia. Both of the parameters detect different aspects of balanced general anesthesia [14]. Park et al. used nasal PPG waveforms for analgesia detection during general anesthesia. This study proposed the nasal PPG index used to predict the pain level during anesthesia with an AUC of 0.73 [15].

To summarize, earlier studies did not comprehensively investigate the key waveform features of PPG in the detection of anesthesia drug detection in the patient. However, some studies tried to improve the accuracy in detecting the depth of anesthesia or balanced anesthesia but still lack a comprehensive comparison of different PPG waveform features, which plays a key role in the development of algorithms to reliably and accurately detect anesthesia in different subjects [12–14]. A similarity in previous studies was found during the literature review that all previous studies only recruit patients having anesthesia only. The comparison between anesthesia and no-anesthesia volunteers could reveal more details of the PPG-based anesthesia detection and provide the true sensitivity of the predicting algorithm. To make a reliable anesthesia depth detection algorithm from the PPG waveform, a machine learning classifier trained with significant waveform features is needed.

This pilot study was designed to investigate the key PPG waveform features and develop machine learning classifiers to achieve reliable PPG-based anesthesia drug detection in the patients. Multiple classification algorithms were developed, trained, and evaluated based on the data from both anesthesia and non-anesthesia patients.

## Materials and methods

**Fig. 1:**
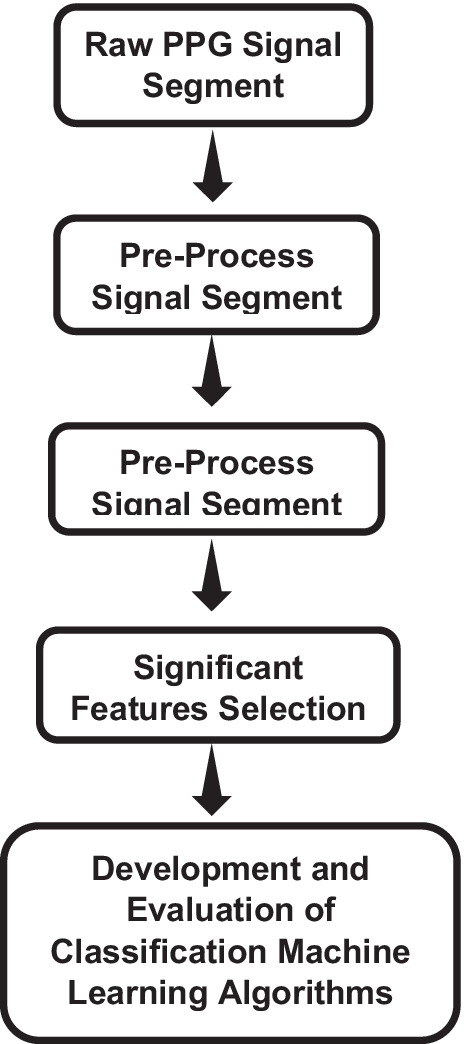
Flow diagram of the study

The major steps of this pilot study are summarized in Fig. 1:

1. Extract and segment raw PPG waveforms from two online databases (Queensland and MIMIC-II). Based on the manual check, the good-quality signal segments with 5 s of duration were saved.
2. Pre-process each PPG waveform segment using noise filtration, baseline elimination, and 2-dimensional (2D) normalization (*x*-axis and *y*-axis).
3. Extract and label PPG waveform features.
4. Identify the significant features affected by the anesthesia drugs using a *t*-test.
5. Train and evaluate classification algorithms using tenfold cross-validation and compare anesthesia detection accuracy.

### Physiological signal databases

In this study, two online physiological signal databases (Queensland and MIMIC-II) were used to extract PPG waveforms:

#### Queensland database

**Fig. 2:**
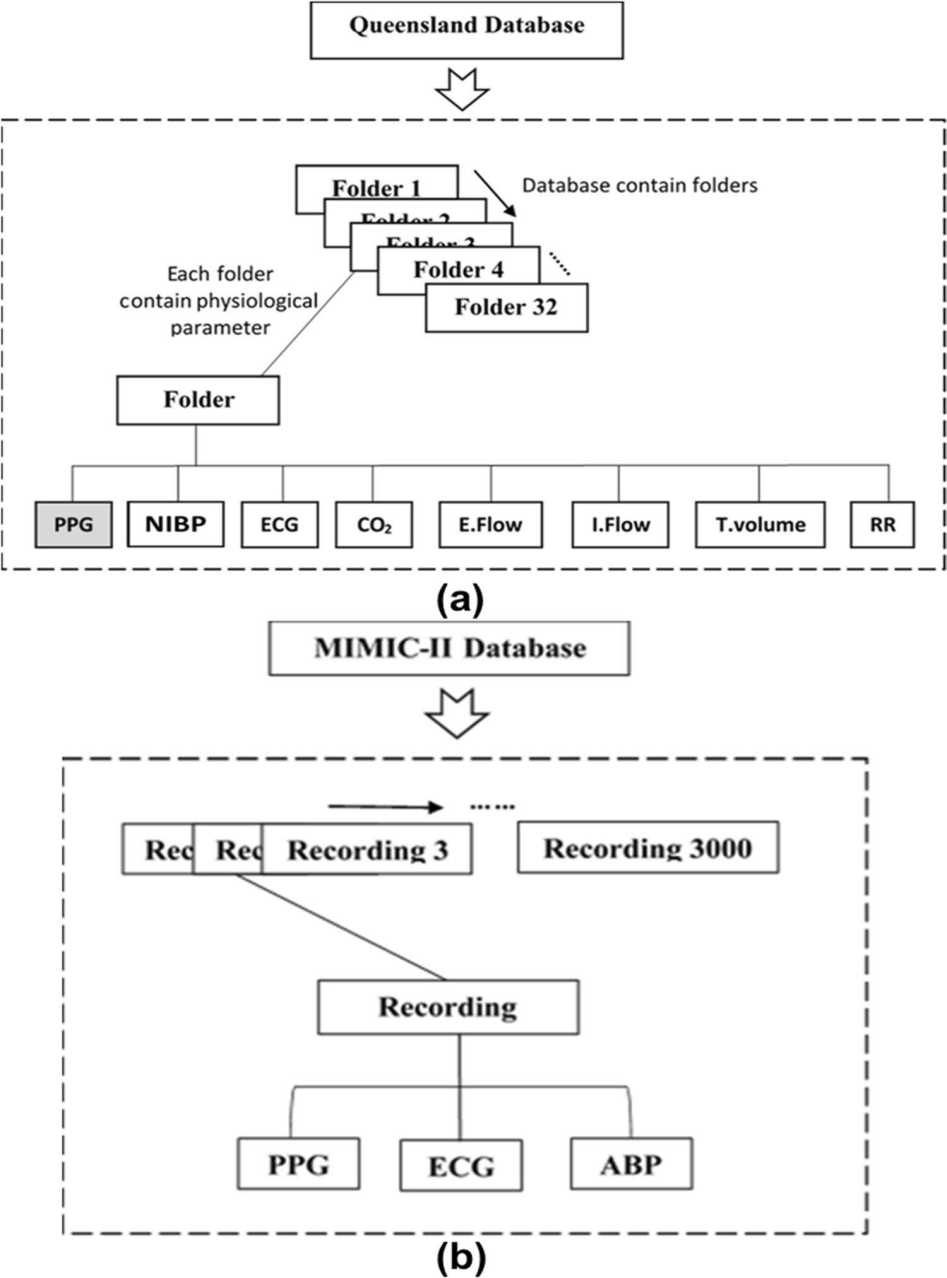
Physiological databases. **a** Queensland database structure which consists of 32 folders; each folder contains eight physiological parameters. Only PPG signal recordings were separated (shaded box) for further processing. **b** The MIMIC database contains 3000 recordings of three physiological parameters, including PPG signals. Each recording folder contains recorded parameters from each non-anesthesia and anesthesia patient with variable measurement duration

Queensland database contains pre-recorded physiological parameter data, including PPG waveforms from patients who had anesthesia as shown in Fig. 2(a) [16]. These signal recordings were affected by the movement artefacts and also faced missing data problems. Therefore, signal recordings were segmented into 5-s signal segments. The Queensland database contains PPG waveforms from 32 patients who underwent surgical procedures (25 for general surgery, 4 for sedation, and 3 for spinal anesthesia). With variations of signal quality among the 32 cases, finally, 8155 5-s PPG signal segments were extracted in total. Each folder contained excel files that have multiple physiological signal recordings with variable measurement duration. The PPG signals were recorded using a 100-Hz sampling frequency.

#### MIMIC II database

Similarly, only 32 cases were randomly separated from 3000 patient data in the MIMIC II database. To further match the data sizes of anesthesia and non-anesthesia patients, 8155 5-s PPG signal segments were extracted from the 32 recordings. Each recording contains three physiological parameter data, as shown in Fig. 2(b). All physiological waveforms were recorded with a 125-Hz sampling frequency. In total, 16,310 PPG waveform segments were used; each sample contained five average PPG features from the beats present in each PPG waveform segment.

### PPG signal pre-processing

**Fig. 3:**
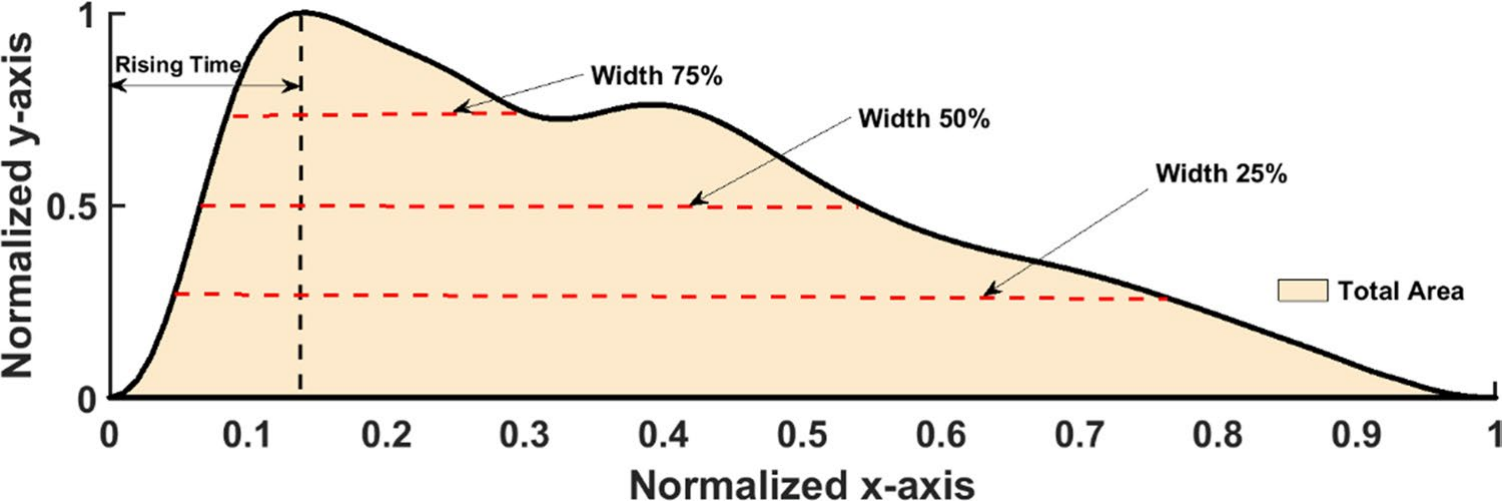
Extracted PPG waveform features from every single beat of the PPG waveform extracted from a 5-s signal segment. The rising time is indicated by the vertical black dashed line which points to the time on the horizontal axis, widths are indicated by the horizontal red dashed lines, and the total area is represented by the creamy corn color under the curve

Savtizky-Golay filter was preferred over other averaging filters as it preserves the edges of the PPG waveform [17]. In this study, a filter having 4 poles and 20 frames was used to maximally reduce the noises. The baseline wandering caused by the respiratory drive was removed from each PPG waveform segment. After the removal of baseline wandering and high-frequency noises (e.g., powerline noise, electromyographic noise, motion artefact), the 2D normalization (in both width and amplitude) was then performed on each cardiac cycle (i.e., pulse beat) to protect PPG waveform segments from amplitude and sample rate variation between two PPG waveform databases [8, 9]. A pre-processed pulse is depicted in Fig. 3.

### PPG waveform features extraction

In this study, only key signal features (total area, rising time, width 75%, 50%, and 25%) that have significant point-biserial correlation > 0.6 with anesthesia drug were selected from 13 PPG signal waveform features (rising time, total area, width 10% [i.e., the width of the waveform at 10% of the total amplitude], width 20%, width 25%, width 30%, width 40%, width 50%, width 60%, width 70%, width 75%, width 80%, and width 90%). The selected key signal features were used and discussed in this study that was extracted from each beat of the PPG waveform segment, as shown in Fig. 3. These PPG pulse features are associated with vascular tone, BP changes, and systemic vascular resistance [18, 19]. Rising time is the duration between the onset and the peak point of the PPG signal pulse, as shown in Fig. 3. The rising time has been used as a key feature to detect cardiovascular irregularities [20]. The total area under the PPG signal waveform was calculated by dividing the waveform space into N equal trapezoid triangles. The area under the curve has a significant relationship with the BP [21]. The pulse widths at different heights were calculated against the normalized *y*-axis. It has been reported that PPG pulse width has a strong relationship with systemic vascular resistance [22].

The detection of peak and onset points was based on the slope (i.e., first derivative) of the PPG signal waveform. The algorithm detects the extrema when the sign of slope changes (i.e., from positive to negative for maxima, from negative to positive for minima), from which the peak and onset were selected as the global maximal and minimal values. Based on the peak and onset, the height (rising time), area, and widths were calculated in each beat.

### Machine learning algorithms

Three classification machine learning algorithms were evaluated in this study for the detection of anesthesia and non-anesthesia patients from PPG signal features only. The algorithm details are as follows:

#### Discriminant analysis

**Fig. 4:**
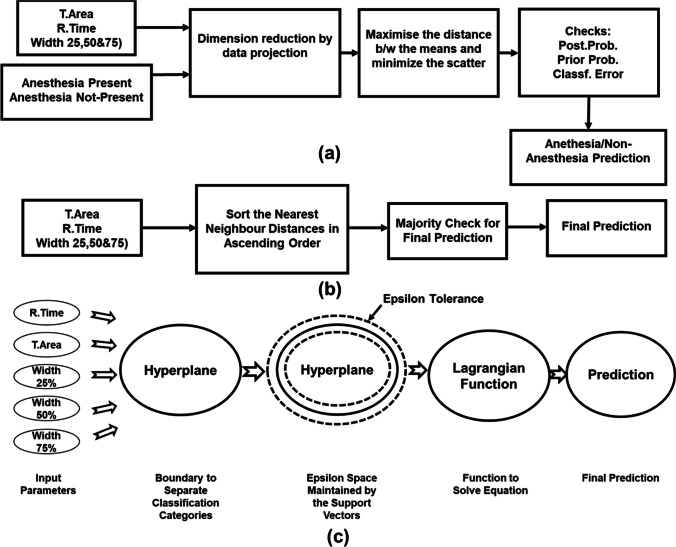
Flow diagrams of supervised classification machine learning algorithms. **a** Simplified diagram of discriminant analysis algorithm used to predict anesthesia drugs present in the human body. The algorithm used means and covariance in dimension reduction to increase the distance between the means and scatter. The last step checked the posterior probability, prior probability, and classification error before prediction. **b** The KNN algorithm analyzes PPG waveform features and measures Minkowski distances between the query point and three neighboring data points. Later, a majority check was performed to finalize the detection result of anesthesia/non-anesthesia patients. **c** A flow diagram of the SVM algorithm that shows the hyperplane and epsilon tolerance (surrounded by dashed lines) created by the support vectors

In this research, a linear discriminant analysis classifier was used for anesthesia drug detection. This algorithm uses five sets of PPG waveform features present in each row of 16,310 total samples. This algorithm reduced dimension by data projection. It maximizes the distance between means and reduces scatter. Next, it checks prior and posterior probability and predicts the outcome, which is anesthesia or non-anesthesia patients [23]. The summary of discriminant analysis algorithm actions is labeled in Fig. 4(a).

In this algorithm, anesthesia detection is based on the least classification cost in Eq. 1:

1 $$\widehat{y}=\mathrm{arg min}{\sum }_{k=1}^{K}P\left(k|x\right)C(y|k)$$

where:

ŷ = detection.

K = number of categories (anesthesia/non-anesthesia).

P(k|x) = posterior probability.

C(y|k) = classification error of extracted signal features.

#### K-nearest neighbor

KNN is the widely followed classification technique among researchers. It measures the distances between a query and neighboring points in the training data. All the distances were sorted in ascending order, and, based on majority vote criteria, predicted the anesthesia drugs in the patient from PPG waveform features [24]. A summary of the necessary steps of the KNN algorithm for anesthesia patient detection is shown in Fig. 4(b).

In the KNN algorithm, the distance between the query point and the neighboring data points measure Euclidean distance and standardize Euclidean distance, Mahalanobis distance, city block distance, Minkowski distance, Chebychev distance, cosine distance, correlation distance, Hamming distance, Jaccard distance, and Spearman distance. In this study, Minkowski distance with *K* = 6 neighboring data points was used to train and test the anesthesia detection algorithm. Malinoski distance uses the following Eq. 2 for the metric:

2 $${d}_{st}= \sqrt[p]{{\sum}_{j=1}^{n}|{x}_{sj}-{y}_{tj}{|}^{p}}$$

This KNN algorithm develops to find the closest *x* point to set the *y* point.

#### Support vector machine

This algorithm selects the most appropriate hyperplane that separates the data into different categories. The hyperplane selection is based on the largest margin between different categories. It uses the Lagrangian function to separate the PPG waveform features of anesthesia and non-anesthesia categories based on the epsilon tolerance developed from support vectors. The linear kernel was used in the training of this algorithm. A simplified block diagram of all the necessary step SVM algorithm to detect anesthesia is shown in Fig. 4(c). The algorithm becomes converged when the feasibility gap tolerance between the primal and dual objective function is less than 1 e-3 [25].

In this research, only two categories (anesthesia/non-anesthesia) are available to differentiate. The best hyperplane between these categories consists of the largest possible margin between the categories. In this classification algorithm, the training data set is represented as xj and predicted variables (yj). The primal Eq. (3) of the hyperplane formation is:

3 $$f\left(x\right)={x}^{^{\prime}}\beta +b=0$$

where β ϵ Rd and b represent the real number; this equation implies a suitable hyperplane for identifying anesthesia and non-anesthesia. Here, β and b would be reduced for all the data points (xj, yj)

4 $${y}_{j }f\left({x}_{j}\right) \ge 1$$

The support vectors present on the decision boundary are x_j_, and the others:

5 $${y}_{j }f\left({x}_{j}\right)=1$$

### Data processing

In total, 13 PPG signals were analyzed using the statistical point biserial correlation method to quantitatively assess the relationship between PPG signal features and anesthesia drugs [26]. Only 5 key features were selected that showed a correlation higher than 0.6. For each feature, the values extracted from different beats were averaged to derive a single estimation for the 5-s segment for next-step analysis.

The dataset that consists of 16,310 data segments of 64 patients was divided into two halves. The first half which consists of 8570 PPG signal segments (32 patients, 16 anesthesia, and 16 non-anesthesia) was used for algorithm training. The other half which consists of 7740 segments (32 patients, 16 anesthesia, and 16 non-anesthesia) was used for algorithm testing.

For this study, the normality test was not performed since the sample size far exceeded the sample size limit of 40 [27]. It is suggested that a parametric procedure can be used even if the data is non-parametric when the sample size exceeds the 40-sample limit [27].

The box plot was used to compare the feature segments extracted from anesthesia and non-anesthesia patients (i.e., data from Queensland and MIMIC II datasets). The *t*-test was applied to check if there was any significant difference in PPG features between anesthesia and non-anesthesia patients. The key features were separated for machine learning algorithm development.

The tenfold cross-validation was used during each iteration of optimization of machine learning algorithms. Each data segment was labeled with a patient number in order to avoid any bias between the training and testing steps of cross-validation. The optimized algorithms were applied to the testing dataset to check their performance.

The confusion matrix was created to calculate error rate, sensitivity, false positive rate, specificity, precision, and Cohen’s kappa. The region of interest (ROC) curves were used to check area under the curve (AUC) of each algorithm.

## Results

**Fig. 5:**
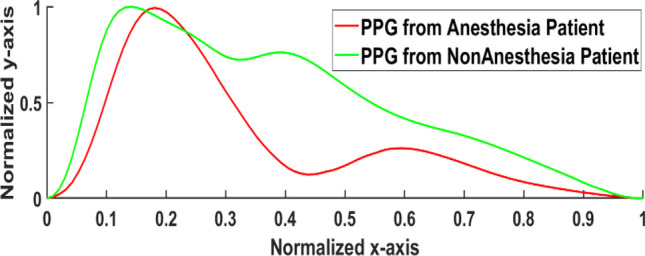
The average and normalized PPG waveform waveforms of anesthesia and non-anesthesia patients admitted to the hospital

Figure 5 shows the normalized PPG waveforms of the patients having anesthesia drugs (red line) and the non-anesthesia patients (green line). Both PPG waveforms have different wave characteristics in terms of time-based waveform features (peak timing, area under the curves, and widths).

### The t-test results

**Fig. 6:**
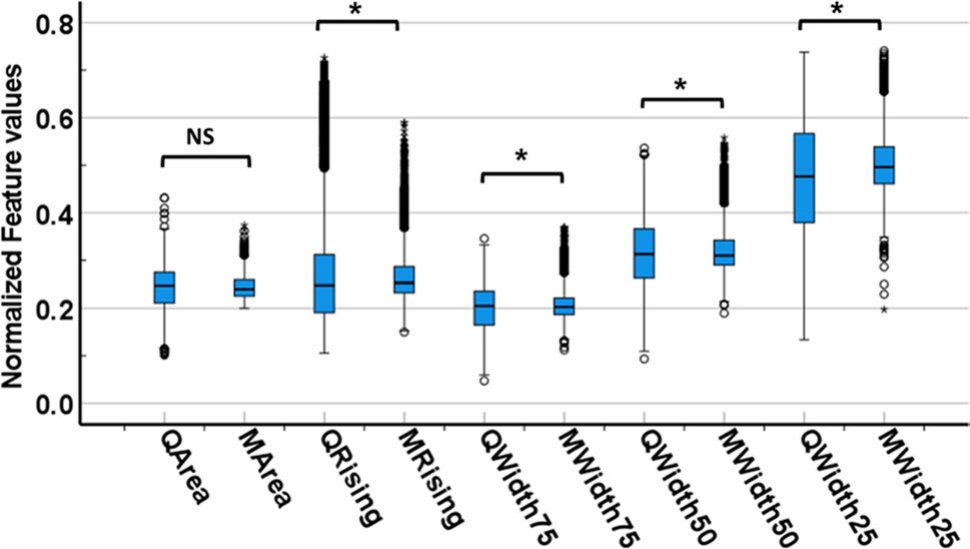
The box plot diagram of PPG waveform parameters extracted from anesthesia (features name starting with Q for Queensland database) and non-anesthesia (M for MIMIC II database) patient’s PPG waveforms. The values of each PPG waveform feature were compared between anesthesia and non-anesthesia patient subgroups. Note: * denotes a significant difference and NS means no significant difference

The two-sample unequal variance *t*-test was applied to the time-based PPG waveform features extracted from anesthesia (i.e., from the Queensland database) and non-anesthesia patients (i.e., from MIMIC II database). This *t*-test aims to identify the significant parameters for the training and testing of three classifiers (discriminant analysis, SVM, and KNN). The *t*-test results’ identified total area feature has no significant difference between the anesthesia and non-anesthesia patients (*p* > 0.05). Conversely, other waveform features (rising time, width 75%, width 50%, and width 25%) show significant difference (*p*-value < 0.05) in Fig. 6.

### Classifier optimization

**Fig. 7:**
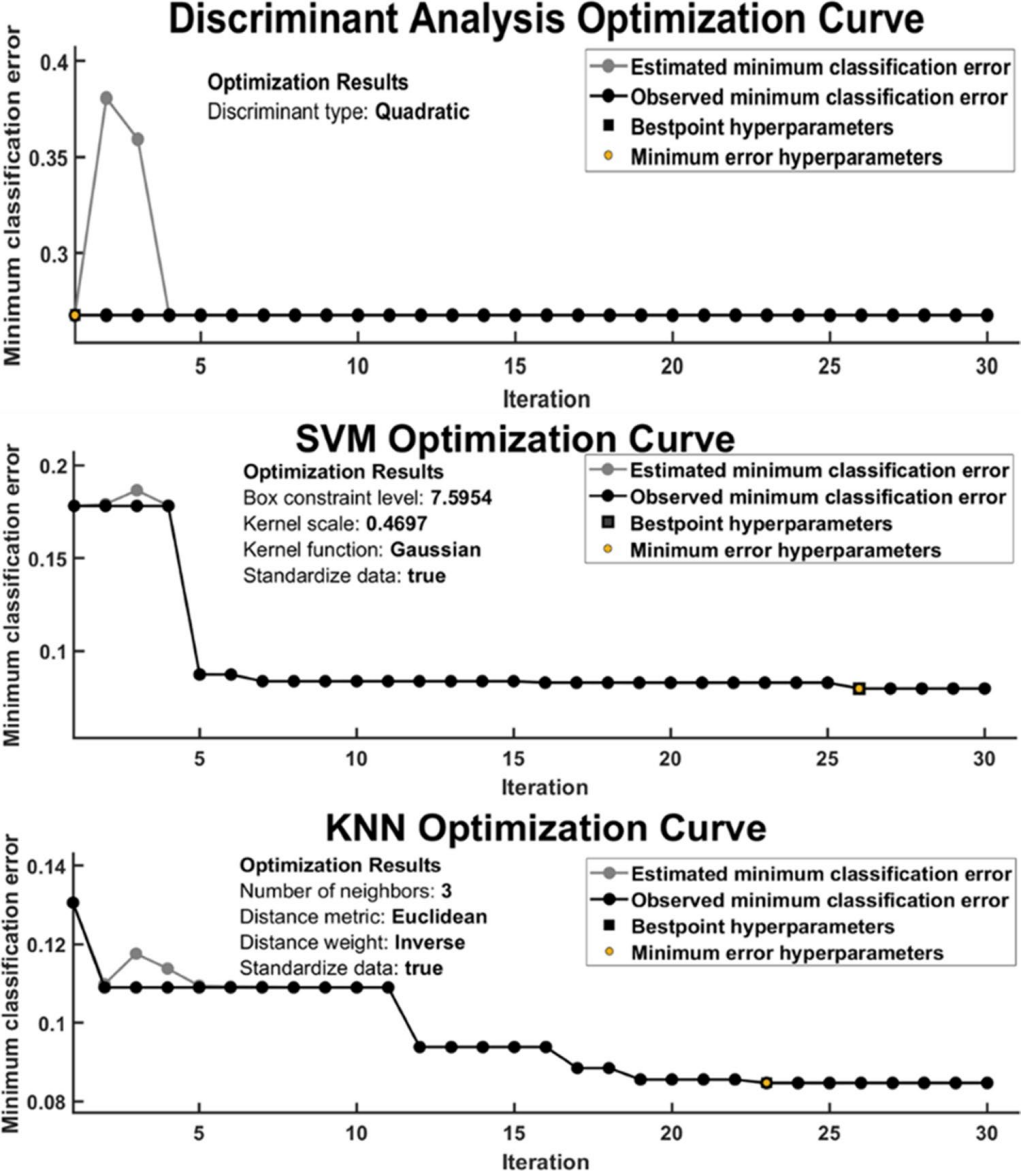
Optimization curve of the classifiers shows estimated and observed minimum classification errors in 30 iterations under tenfold cross-validation

The Bayesian optimizer algorithm was used to optimize all three classifiers to get hyperparameters using tenfold cross-validation. All three classifiers (discriminant analysis, SVM, and KNN) were optimized with 30 iterations to find the best hyperparameters having minimum classification error as shown in Fig. 7.

The SVM classifier achieved the best hyper-parameters (box constraints = 0.32 and kernel function = cubic) at the minimum classification error of 0.165 on 26th iteration during optimization. Similarly, the discriminant analysis classifier achieved the best hyperparameters (discriminant type = quadratic) at the minimum classification error of 0.29 on the 1st iteration. At last, the KNN classifier achieved the best hyperparameter (number of neighbors = 3; distance metrics = Euclidean, and distance weight = inverse) at the minimum classification rate of 0.09 on the 23rd iteration during optimization.

### Classifiers evaluation

Among highly optimized classifiers, KNN classifiers achieved the highest anesthesia detection accuracy (91.7%) as shown in Table 1, whereas SVM and discriminant analysis classifiers achieved accuracies even less than 75% (66.5% and 73.4).

**Table 1:** Anesthesia detection accuracies of machine learning classifiers tested using testing dataset

Classifiers	Hyperparameters	Detection accuracy (%)
Discriminant analysis	Discriminant type = quadratic	73.4
SVM	Box constraints = 7.59 Kernel function = Gaussian	66.5
KNN	Number of neighbors = 3 Distance metrics = Euclidean Distance weight = inverse	**91.7**

**Fig. 8:**
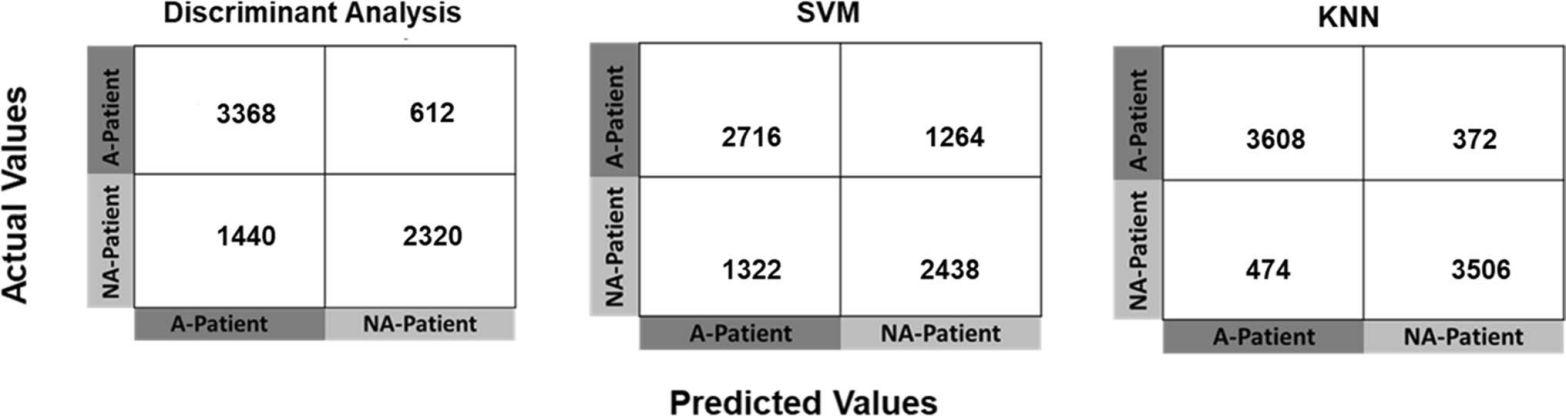
The Confusion matrices of three classifiers (discriminant analysis, SVM, and KNN) trained and tested with four key PPG waveform features (rising time, width 75%, width 50%, and width 25%) using the split into two halves technique. Note: A, anesthesia; NA, non-anesthesia

Furthermore, the confusion matrices of the classifiers show the details of how these classifiers perform to successfully classify patients having anesthesia using significant PPG waveform features only. The numbers in the confusion matrices show KNN outperformed and separate the highest number of anesthesia and non-anesthesia PPG features (3608 and 3506) among all the classifiers under test as shown in Fig. 8.

**Fig. 9:**
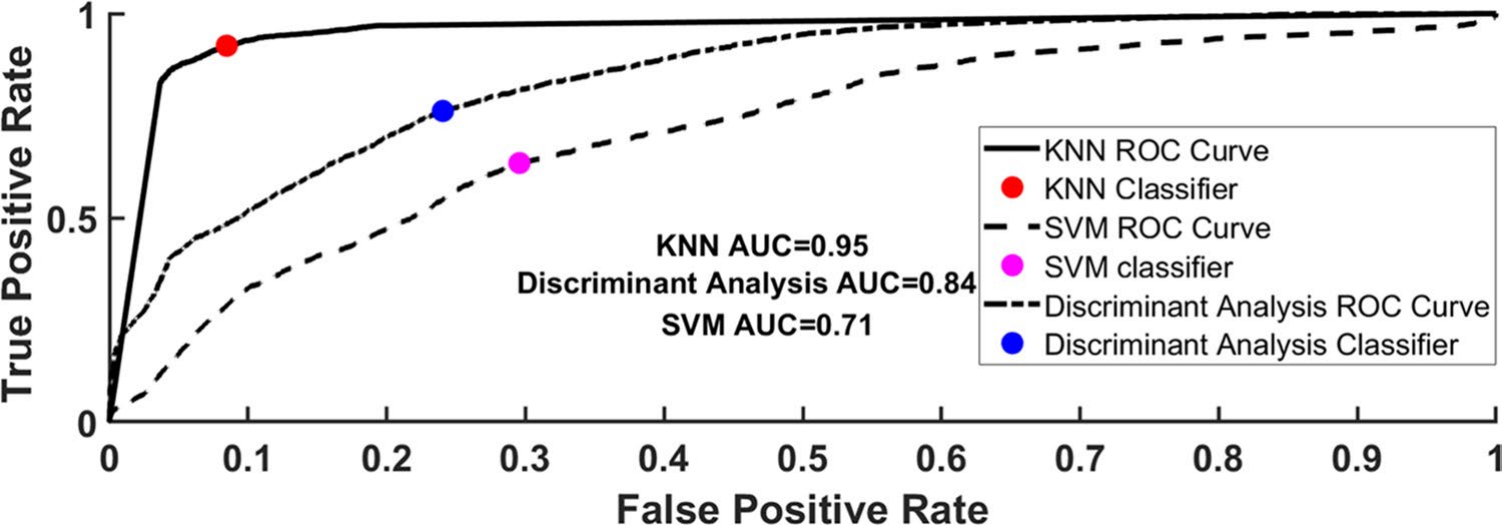
The area under the curve (AUC) of all classifiers (discriminant analysis, SVM, and KNN) in the detection of the patient having anesthesia drug-using significant PPG features. The KNN achieve the best AUC of 0.95 with the highest sensitivity of 0.88 and the least false-positive rate of 0.09

In Fig. 9, the area under the curve of all three classifiers for the classification of patients having anesthesia drugs and no-anesthesia shows that the KNN classifier achieves the best AUC of 0.95 as compared to discriminant analysis (0.84) and SVM (0.71) classifiers.

Moreover, the KNN classifier achieved a low error rate (0.10) against discriminant analysis (0.26) and SVM (0.33). The sensitivity (0.88), specificity (0.90), and precision (0.90) also show the effectiveness of the classifier using significant PPG waveform features in Fig. 6. To determine the inter-rater reliability of the classifier under test, Cohen’s kappa coefficient was calculated that shows KNN almost has a perfect agreement (0.79) or inter-rater reliability as compared to other classifiers which show the fair agreement (0.46 and 0.33) as listed in Table 2.

**Table 2:** Calculated parameters from confusion matrices where KNN achieved better performance among all the classifiers

Calculated parameters	Discriminant analysis	SVM	KNN
Error rate	0.26	0.33	**0.10**
Sensitivity	0.61	0.64	**0.88**
False positive rate	0.15	0.31	**0.09**
Specificity	0.84	0.68	**0.90**
Precision	0.79	0.65	**0.90**
Cohen’s kappa	0.46	0.33	**0.79**

## Discussion

This study shed light on reliable PPG-based anesthesia drug detection. Following existing studies, the results confirmed the potential of PPG waveform significant features and machine learning classifiers in detecting anesthesia drugs. Based on the quantitative comparison between different waveform features, the KNN algorithm achieved high accuracy of detection.

### Significance of PPG waveform features in anesthesia depth detection

Generally, PPG has been used during anesthesia monitoring to monitor vital signs (heart rate) and oxygen saturation only. This study will open a new chapter toward reliable anesthesia drug monitoring with the machine learning classifier using significant time-based PPG waveform features. A previous study also mentioned the relationship between PPG and nociception (it is a process of the sensory nervous system that encodes the noxious substance) [14]. Another study used PPG acceleration features to determine the depth of anesthesia and compared it with the cerebral state index, which was also proposed for anesthesia monitoring [15]. Some other studies found the relationship between PPG and anesthesia, but those studies are only pilot studies that were carried out with a small amount of data.

None of the studies comprehensively investigated and identified PPG waveform features that have high sensitivity to differentiate between PPG having anesthesia drugs and non-anesthesia. Although some research groups attempted to evaluate their PPG-based technologies on anesthesia patients and achieved maximal AUC 0.91 [11], the current machine learning classifier (KNN) using significant PPG features achieved AUC 0.95 as listed in Table 3. Nevertheless, it should be noted that our comparison was between anesthesia and non-anesthesia status, not between different depths of anesthesia as in some existing studies. The performance of our algorithm in the fine-grained classification of different anesthesia conditions deserves further investigation.

**Table 3:** Comparison between earlier research and our study

Author	Source	Parameters	Method	Number of participants
Coutrot et al.2019	Finger PPG	DIC + PI	Statistical analyses (no machine learning)	61 (all under anesthesia)
Ezri.T et al.1998	Finger PPG	Amplitude	PPG amplitude proportionality	50 (all under anesthesia)
Chen W et al. 2020	Finger PPG	Approximate and sample entropies	Prediction probability	40 (all under anesthesia)
Bao.H. et al	Finger PPG	Amplitude, notch, baseline amplitude, and area	Prediction probability	45 (all under anesthesia)
Park C et al. 2020	Nasal PPG	Nasal photoplethysmography index	Statistical analyses	81 (all under anesthesia)
Roy Chowdhury et al., 2021	ECG and Finger PPG	ECG and PPG heatmaps	Deep learning	50 (all under anesthesia)
**Our Work**	**Finger PPG**	**Rising T, width 75%, width 50%, width 25%**	**KNN classifier**	**64 (32 = anesthesia, 32 = non-anesthesia)**

### PPG signal quality evaluation

The PPG signal quality is influenced by many factors including environmental (i.e., ambient light intensity), technical (e.g., sensor layout, skin attachment, powerline noise, hardware for denoising), and physiological (e.g., body movement, skin color, measurement site) factors [28]. Many methods have been proposed for PPG signal quality evaluation and processing based on morphological, spectral, and statistical characteristics [29]. Due to the high heterogeneity of PPG signal quality, there is a lack of a standardized method for PPG signal quality assessment algorithm and manual assessment is still widely adopted as a reliable reference [30]. In this study, the Queensland database contains electronic glitches and movement artefacts found in various places. Therefore, we systematically analyze the data manually and concluded that the 5-s signal segment which consists of 4 cardiac cycles (0.8 s cardiac cycle × 5 s) not affected by the noise.

### Machine learning algorithms for anesthesia depth detection

Previous studies also lack the development of a reliable and accurate machine learning classifier. A recent study used both ECG and PPG signal heatmaps for the training and evaluation of deep learning algorithms to predict the depth of anesthesia. Consequently, no time-based features were discussed as the whole input signal was converted into heatmaps [6]. Many researchers extracted EEG signal features for training and validation of different machine learning algorithms (fine decision tree, artificial neural network, and SVM) to get better accuracy (> 90%) in anesthesia depth monitoring but EEG has more complex signal waveforms than PPG [31–33]. Furthermore, previous studies lack detailed algorithm evaluation.

To summarize, no earlier study comprehensively evaluated and applied the time-based PPG features in the machine learning-based classification of anesthesia and non-anesthesia patients, as summarized in Table 3. Therefore, there was a need for such a study that could investigate the significance of key PPG waveform features using updated machine learning techniques to better analyze classifier sensitivity in the identification of patients having anesthesia drugs. The waveform features selected in this study for the detection of anesthesia drugs within the patient have been identified as the important markers for cardiovascular and pulmonary activities that have a major role during anesthesia depth monitoring. This study will provide a platform for further research about the determination of reliable and accurate anesthesia depth detection, monitoring of individual anesthesia drugs during surgery, and post-operative monitoring of the patients having sedative pills.

### Study limitations

This study is limited to a combined dataset of 64 patients from Queensland and MIMIC-II (32 patients each) databases as it specifically investigated the potential of PPG application in the detection of non-invasive anesthesia drugs in the patients. The demographic data of the patients were not included in the dataset, therefore not considered in this study. It is well known that the morphology of PPG is affected by these factors (e.g., age, sex, body mass index). No anesthesia depth reference (BIS or spectral entropy) was available in the investigated online databases (Queensland and MIMIC-II).

### Future work

In the future study, an extensive dataset including demographic and clinical data will be created using hospital databases along with the anesthesia depth reference (BIS) to develop a better machine learning model that could estimate the correct depth of anesthesia and provide a reliable and cost-effective monitoring solution to the staff. Additionally, deep learning has been proposed as a promising tool to achieve reliable detection of anesthesia, which deserves exploration based on multi-center, large-scale datasets [34].

## Conclusion

This study shows the significant potential of a temporal PPG markers-based machine learning classifier as a key step toward accurate anesthesia depth detection. It paves the way for the development of PPG-based reliable algorithms toward fine-grained anesthesia depth analysis. Furthermore, PPG can also be used to determine the effects of anesthesia drugs on the human body during the drug trials as it is not sensitive to any anesthesia drug. This research also lays the groundwork for the development of a handheld and low-cost portable device for the monitoring of anesthesia drugs during surgical operations and post-operation within the hospital.

## Data Availability

Data can be accessed via these links: 1) https://outbox.eait.uq.edu.au/uqdliu3/uqvitalsignsdataset/index.html. 2) https://archive.physionet.org/mimic2/

## Code availability

The code is restricted due to the patent application (WO2021038237A1).
